# Supplementary figures and images for: Identification and characterization of cold-responsive aquaporins from the larvae of a crambid pest Agriphila aeneociliella (Eversmann) (Lepidoptera: Crambidae)
Source: PeerJ. 2023 Nov 13;11:e16403. doi: 10.7717/peerj.16403 (PMC10652857; doi:10.7717/peerj.16403)

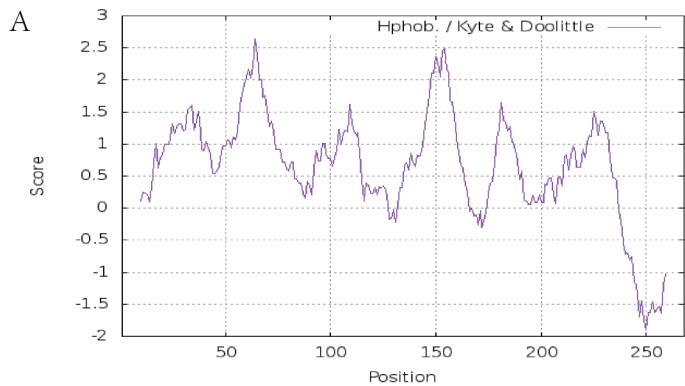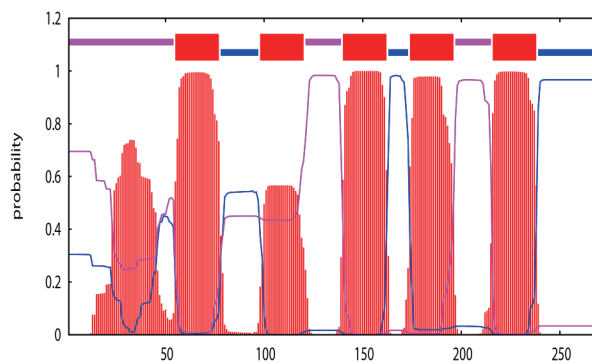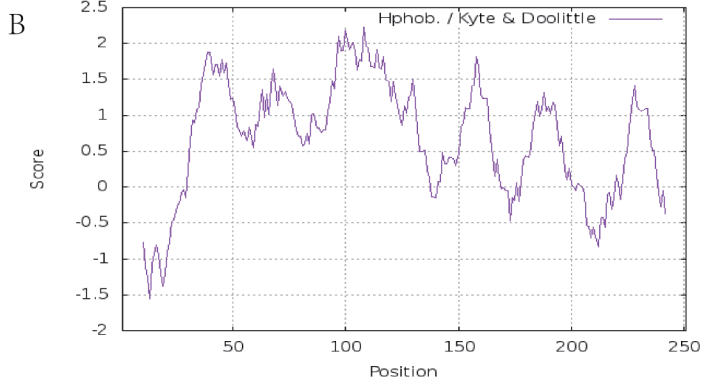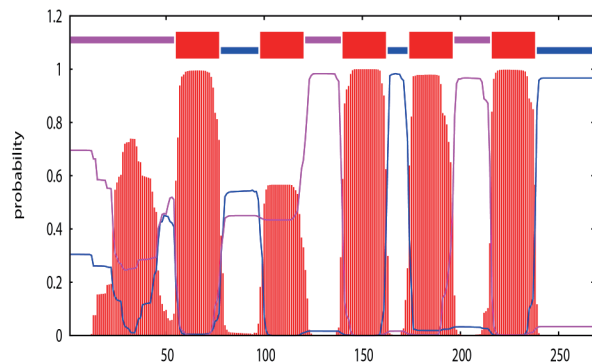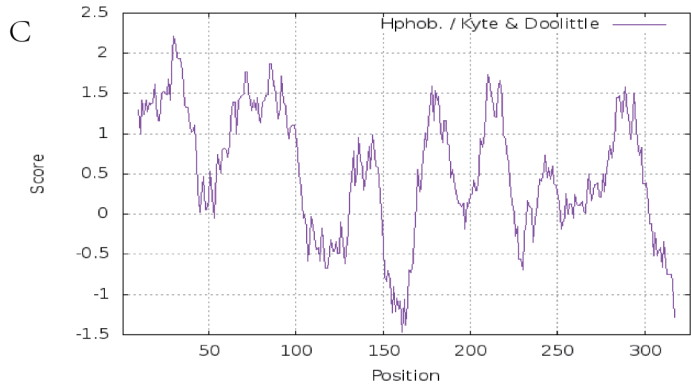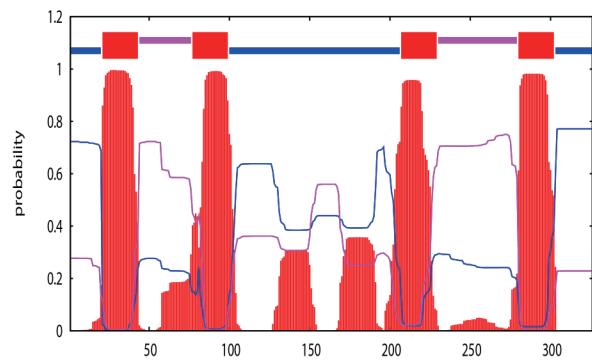

Supplement: Supplemental Information 1 — The results of representative sequence of classical aquaporins (A), aquaglyceroporins (B) and AQP12L (C) are as shown. ExPASy (http://us.expasy.org/tools/) and TMHMM tools (http://www.cbs.dut.dk/services/TMHMM/) are used in this analysis. Rransmembrane domains are comprised by using red lines, inside and outside of the cell membrane are marked with blue and pink lines, respectively [file peerj-11-16403-s001.pdf]
