# Supplementary material for: Identification and characterization of cold-responsive aquaporins from the larvae of a crambid pest Agriphila aeneociliella (Eversmann) (Lepidoptera: Crambidae)
Source: PeerJ. 2023 Nov 13;11:e16403. doi: 10.7717/peerj.16403 (PMC10652857; doi:10.7717/peerj.16403)
Supplement: Supplemental Information 2 [file peerj-11-16403-s002.docx]

Table S1 Primers used in this study

| Primer names | Primer sequences (5’-3’) | Purpose |
| --- | --- | --- |
| AaAQP1-F  AaAQP1-R | GTGTTCGTGCCTTGGTCTAGTTG  CCAGGTCTTCAGTCTTACCGTCT | ORF cloning |
| AaAQP2-F  AaAQP2-R | CTGCGGTCGGATTCTGTCAAATA  TGCGGAACACGAACCTGTAGAGTAG |  |
| AaAQP3-F  AaAQP3-R | GCTAACTACATCGAGAACACCCA  GACAATAGGATTTCAATGCCAACAG |  |
| AaAQP4-F  AaAQP4-R | ACATCCTCCTCGTCCTATCATCA  GCTTTAGGGGTAGAAATCGTGCT |  |
| AaAQP5-F  AaAQP5-R | TACAGGCAGTGTTATGGGGAAGA  ACTTGTAGAATATGGCCGGAATG |  |
| AaAQP6-F  AaAQP6-R | ACGGATTACGCTGTGGATGAGAT  CGGGAGATTTGTCGGAAACACTA |  |
| AaAQP1-F  AaAQP1-R | TCAGGCGGACATATCAACCC  CAGGACGAATCCAAGGAAGAAT | RT-qPCR |
| AaAQP2-F  AaAQP2-R | ACGGATTACGCTGTGGATGAG  GCAAGTCACTGCTGGGTTTATG |  |
| AaAQP3-F  AaAQP3-R | AGGCAGTGTTATGGGGAAGAA  ACCTCCAATGGTAAGGGCAT |  |
| AaAQP4-F  AaAQP4-R | CAGCGACGATGTTCCTGATT  CCTTCTCCACCCATGTCAACT |  |
| AaAQP5-F  AaAQP5-R | GTAGGCTTCGGCTTATTGTCC  GGCACGGTCGGGTTTGTC |  |
| AaAQP6-F  AaAQP6-R | GCTGGTGGCGGAGTTGAT  TGACGAGCACGACGAAGC |  |
| *β-actin*-F | TACTCCGTATGGATCGGTGGATC |  |
| *β-actin*-R | TTAGAAGCACTTGCGGTGGAC |  |
